# Characterization by Gene Expression Analysis of Two Groups of Dopaminergic Cells Isolated from the Mouse Olfactory Bulb

**DOI:** 10.3390/biology12030367

**Published:** 2023-02-25

**Authors:** Fabio Casciano, Nicoletta Bianchi, Mirta Borin, Vittorio Vellani, Paola Secchiero, Carlo M. Bergamini, Simona Capsoni, Angela Pignatelli

**Affiliations:** 1 Department of Translational Medicine and LTTA Centre, University of Ferrara, 44121 Ferrara, Italy; 2 Interdepartmental Research Center for the Study of Multiple Sclerosis and Inflammatory and Degenerative Diseases of the Nervous System, University of Ferrara, 44121 Ferrara, Italy; 3 Department of Translational Medicine, University of Ferrara, 44121 Ferrara, Italy; 4 Department of Neuroscience and Rehabilitation, University of Ferrara, 44121 Ferrara, Italy; 5 Department of Biomedical, Metabolic and Neural Sciences, University of Modena and Reggio Emilia, 41125 Modena, Italy; 6 Bio@SNS Laboratory of Biology, Scuola Normale Superiore, 56126 Pisa, Italy

**Keywords:** olfactory bulb, adult neurogenesis, dopamine, FACS, gene expression

## Abstract

**Simple Summary:**

In the olfactory bulb, dopaminergic cells keep being replaced throughout life in all mammalians, including humans. These neurons can be easily identified in transgenic mice expressing eGFP under the tyrosine hydroxylase promoter, as they become fluorescent, and can also be isolated from other neurons using enzymatic dissociation and fluorescence-activated cell sorting (FACS), allowing investigation of gene expression in neurons with different levels of fluorescence. We demonstrated for the first time that fluorescence intensity correlates very well with the expression of genes typical of different stages of maturation in dopaminergic neurons. We propose that the FACS method used to isolate these neurons may be used to engineer new neurons for therapeutic purposes in widespread pathologies, such as Parkinson’s disease.

**Abstract:**

The olfactory bulb (OB) is one of two regions of the mammalian brain which undergo continuous neuronal replacement during adulthood. A significant fraction of the cells added in adulthood to the bulbar circuitry is constituted by dopaminergic (DA) neurons. We took advantage of a peculiar property of dopaminergic neurons in transgenic mice expressing eGFP under the tyrosine hydroxylase (TH) promoter: while DA neurons located in the glomerular layer (GL) display full electrophysiological maturation, eGFP+ cells in the mitral layer (ML) show characteristics of immature cells. In addition, they also display a lower fluorescence intensity, possibly reflecting different degrees of maturation. To investigate whether this difference in maturation might be confirmed at the gene expression level, we used a fluorescence-activated cell sorting technique on enzymatically dissociated cells of the OB. The cells were divided into two groups based on their level of fluorescence, possibly corresponding to immature ML cells and fully mature DA neurons from the GL. Semiquantitative real-time PCR was performed to detect the level of expression of genes linked to the degree of maturation of DA neurons. We showed that indeed the cells expressing low eGFP fluorescence are immature neurons. Our method can be further used to explore the differences between these two groups of DA neurons.

## 1. Introduction

The olfactory bulb (OB) is a region of the brain that has a key role in odor processing, organized in a stratified structure formed by different cell types. In particular, the outermost portion of the adult OB, termed the glomerular layer (GL), is characterized by the presence of dopaminergic (DAergic) interneurons [1,2,3]. These cells play a key role in odor processing [4,5,6]. They are responsible for significant activity-dependent plasticity, controlling the release of dopamine [7,8,9,10], which will lead to an adaptation of the olfactory bulbar network to external conditions. They control the local gain of transmitter release from the terminals of the olfactory sensory neurons [11,12,13] and determine the inhibition of lateral glomerular output [14]. For a complete anatomical, functional and electrophysiological description of these neurons, the reader may refer to the reviews by Pignatelli et al. [2,4,15,16,17].

Interestingly, DAergic interneurons have another remarkable feature: they are generated during adult life in rodents as well as in other mammals, including humans [18,19,20,21,22,23,24,25,26]. Adult neurogenesis is the process of constant generation of new neurons occurring in two regions of adult brain: the subgranular zone of the hippocampal dentate gyrus (SGZ), and the subventricular zone (SVZ) lining the lateral ventricle [27]. Neurons born in the SGZ develop into glutamatergic granule neurons, and integrate into the hippocampal circuits [28,29], whereas newborn neurons originating in the SVZ migrate through the rostral migratory stream (RMS) in approximately 15–20 days, and finally reach the OB to replace the pre-existing cells, through a process that is strongly enhanced by odor enrichment and by the abundance of the olfactory input [20,30,31,32,33,34,35,36,37,38,39,40].

In the OB, neurons are generated at low levels in the embryo/neonate, but their rate of production increases dramatically in postnatal/adult life [23,41,42]. The differentiation of adult-generated dopaminergic neurons is regulated by numerous different signals, mostly transcription factors, which play a central role in regulating stem cell dynamics and reprogramming between distinct somatic lineages [43,44,45].

It has been demonstrated that OB dopaminergic neurons found in the GL express TH (rate-limiting enzyme of the catecholaminergic pathway leading to dopamine synthesis) only once they receive glutamatergic excitatory input from olfactory neurons terminals and from the apical dendrites of mitral and tufted cells [30,31,32,33,34,35,36,41].

**Figure 1:**
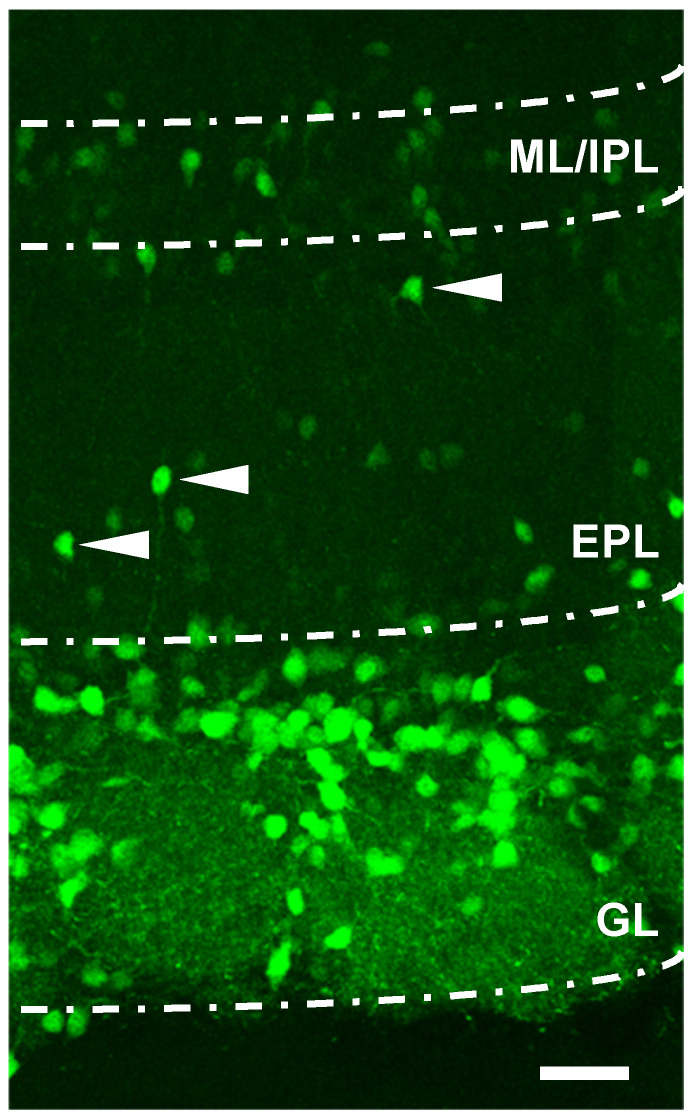
Localization of TH-eGFP+ cells in the olfactory bulb. Confocal image (of 100 μm thickness) slices obtained from TH-eGFP transgenic mice showing the different layers of the olfactory bulb. Scale = 40 μm. Note the different levels of fluorescence expression by the cells in the GL layer with respect to the cells in the M/IPL. The arrows indicate some eGFP+ cells in the EPL layer that are migrating towards the glomerulus. GL (glomerulus layer), EPL (external plexiform layer), and M/IPL (mitral/internal plexiform layer).

More recently, new evidence has indicated that mRNA TH expression can also be found in deeper portions of the OB, including the external plexiform (EPL), mitral and granular layers (GCL) [2,22,46,47]. This has been discovered by means of transgenic animal models that enabled easily distinguishing neurons expressing enhanced green fluorescent protein (eGFP) under the TH promoter, thus tagging live DAergic neurons with a viable, real-time fluorescent reporter. In addition, by observing an OB slice obtained from a TH-eGFP transgenic animals, it can be shown that the fluorescence level of TH-eGFP neurons in the innermost mitral layer and the internal plexiform layer (ML/IP) is less intense compared to that of the cells in the GL, and could suggest that the production of TH is lower in these faintly fluorescent neurons (Figure 1). Cells with low fluorescence levels can also be observed in the EPL and could indicate migrating cells with different levels of maturation in this layer.

In support of this evidence, it has been shown that in the ML/IP layer, there are cells in which the transcription of the TH gene occurs in the absence of significant translational activity [10,22].

In our research group, to explain the presence of TH transcription in M/IP layer cells, it has been proposed that these cells might be adult newly generated, still immature neurons recently arrived from the rostral migratory stream that are committed to become dopaminergic cells [48]. According to this hypothesis, neurons in the ML/IP layers would represent immature cells that stopped their migration in those layers, waiting for molecular signals from cells of the GL which would allow them to cross the EPL, reach the GL and complete there their maturation as DAergic neurons.

This hypothesis has been tested with different electrophysiological methods [46]. The functional characteristics of the supposed immature TH-eGFP+ neurons located in the ML/IP layers were analyzed and compared with those shown by mature DAergic neurons located in the GL. The result of this work showed that the electrophysiological properties of TH-eGFP+ cells have a maturation gradient that starts from the deeper layers (ML/IP layer), goes through the middle layer (EPL) and ends in the outermost GL, where they complete maturation.

The excitability profiles of mature DAergic neurons in the GL have been described previously [16]. These cells show a pattern of ion currents that typically support an autorhythmic pacemaker activity that is their characteristic signature. On the contrary, TH-eGFP+ neurons in the ML/IP layers were found to be very different: the pacemaker currents are absent and, consequently, none of these cells are autorhythmic, although they are perfectly capable of generating trains of action potentials in response to depolarizing pulses [46]. Autorhythmicity is instead observed in the cells in the EPL.

Furthermore, it has been reported that a high intracellular Cl^–^ concentration is a physiological marker of immature neurons [49]. Experimental evidence shows that TH-eGFP+ neurons in the EPL and ML possess a larger intracellular Cl^–^ concentration than those within the GL, indicating a different degree of neuronal maturity [46].

The existing evidence supporting the maturation axis from the ML/IP layer towards the GL of the TH-eGFP+ cells is based on old studies showing that cells require input from olfactory sensory neurons (OSN) through the olfactory nerve (ON) for the expression of the mature DAergic phenotype [50,51,52,53,54]. Recordings from TH-eGFP+ in the EPL layer, after stimulation of the ON, show a synaptic response, while cells in the ML/IP layer fail to do so. Our group proposed that the establishment of a synaptic contact with the ON is the crucial event required to initiate the achievement of cell differentiation from the immature to the mature state.

Interestingly, the degree of maturation of the dopaminergic neurons is paralleled by the intensity of expression of the reporter protein eGFP [46,55], providing a very effective way to separate dopaminergic cells in groups corresponding to different stages of differentiation using fluorescence-activated cell sorting (FACS). By sorting cells according to the intensity of their fluorescence in TH-eGFP mice, it is then possible to analyze the level of expression of several different genes, obtaining a synoptic view of DA neuron developmental programs.

Expectedly, this outstanding property of bulbar DA neurons has raised significant interest, as the understanding the signaling system addressing the development of DA neurons could likely provide extremely useful cues for the therapies of many neurodegenerative diseases with a dramatic social impact, such as Parkinson’s disease. Indeed, olfactory bulb dopaminergic neurons might be endowed with some advantages with respect to grafts derived from mesencephalic cells: OB dopaminergic progenitors can integrate very well into large circuits [56], they are characterized by autorhythimicity such as neurons from the substantia nigra [16,17,57] and are less susceptible to develop PD-like neurodegeneration [58]. However, unlike hippocampal neurogenesis, the transcriptional cascade controlling olfactory bulb neurogenesis necessary to distinguish immature from mature cells has not yet been fully described. Given the possible applications in regenerative medicine studies, where these cells might represent a source of new dopaminergic autologous cells [22,59,60], we decided to isolate and analyze with new techniques the two subpopulations (mature and immature) of TH-eGFP+ neurons from mouse OB.

In this paper, we check the reliability of our cell sorting method, and test it by analyzing the archetypical molecular mechanisms involved in migration, differentiation, and maintenance in the differentiated state of DA neurons.

## 2. Materials and Methods

### 2.1. Animals and Surgical Procedures

To minimize animal suffering and the number of mice used, we designed appropriately experimental procedures in accordance with the Directive 86/609/EEC on the protection of animals used for experimental and other scientific purposes and approved by the Campus Veterinarian of Ferrara University (D.M. 263/2021-PR). We used a transgenic mice strain (TH-eGFP/21–31), carrying the eGFP transgene under the control of the TH promoter [61,62]. The TH-eGFP strain was maintained as heterozygous by breeding with C57BL/6 J inbred mice. The transgene construct contained the 9.0-kb 5′-flanking region of the rat TH gene, the second intron of the rabbit β-globin gene, cDNA-encoding eGFP, and polyadenylation signals of the rabbit β-globin and simian virus 40 early genes. A total of 36 mice of ages between 30 and 60 days have been used.

Adult mice (30–60 days old) were used to isolate olfactory bulb neurons and obtain single DA cells. Adult mice were deeply anaesthetized (i.p. injection of 60 mg kg^−1^ of sodium pentobarbital), decapitated, and the brain was exposed, chilled with oxygenated artificial cerebrospinal fluid (ACSF), and the olfactory bulbs were dissected. ACSF had the following composition (in millimoles): 125 NaCl, 2.5 KCl, 26 NaHCO_3_, 1.25 NaH_2_PO_4_, 2 CaCl_2_, 1 MgCl_2_, and 15 glucose. Saline was continuously bubbled with 95% O_2_/5% CO_2_; the osmolarity was adjusted at 305 mOsm with glucose. The dissociation protocol of the olfactory bulb consists of enzymatic digestion and mechanical trituration. The method, with minor changes, was previously described by Gustincich et al. and Pignatelli and Belluzzi [63,64]. Shortly after dissecting and slicing the bulbs, small pieces of the preparation were transferred to a solution containing 0.3% protease type XXIII (Sigma) for 30–45 min at 37 °C. The protease activity was then arrested by 0.1% trypsin inhibitor (Sigma) (10 min, 37 °C) and the bulb chunks were triturated using fire-polished Pasteur pipettes of varying gauges.

Two solutions were used for the tissue preparation: a dissecting solution and Tyrode’s solution. The dissecting medium (DM) contained: 82 mM Na_2_SO_4_, 30 mM K_2_SO_4_, 10 mM HEPES, 5 mM MgCl_2_, 10 mM glucose, and 0.001% phenol red indicator; pH was adjusted to 7.4 with NaOH and the solution was continuously bubbled with 100% O_2_. Tyrode’s solution contained 137 mM NaCl, 5.4 mM KCl, 1.8 mM CaCl_2_, 1mM MgCl_2_, 5 mM HEPES, and 20 mM glucose (all reagents were purchased from Sigma Aldrich, St. Louis, MO, USA); the pH was adjusted to 7.4 with NaOH and the solution was continuously bubbled with 100% O_2_.

After extraction from the skull, bulbs were cut into small pieces and transferred in a solution containing DM and 3% protease type XXIII (Sigma Aldrich, St. Louis, MO, USA) for 30–45 min at 37 °C. After enzymatic digestion, the bulbs were transferred in a solution containing DM, 1% bovine serum albumin (Sigma Aldrich, St. Louis, MO, USA) and 1% trypsin inhibitor (Sigma Aldrich, St. Louis, MO, USA) to stop protease activity (10 min at 37 °C). The following mechanical trituration was obtained using home-made fire-polished Pasteur pipettes of varying gauges in Tyrode’s solution.

### 2.2. Sample Preparation for FACS Sorting Analysis

Dissociated cells from OB in Tyrode’s solution were collected by centrifugation (300× *g*, 10 min at room temperature) and resuspended in phosphate-buffered saline pH 7.4 (PBS, Life Technologies Italia, Monza, MB, Italy) supplemented with 0.5% bovine serum albumin (BSA, Sigma Aldrich, St. Louis, MO, USA). Cells were washed three times and resuspended in a final volume of 2 mL of the same solution for sorting separation. Then, a cell strainer with 70 μm nylon mesh was utilized in order to obtain a single cell suspension (BD Falcon, BD Biosciences, San Jose, CA, USA) that was kept in ice until sorted. Ten minutes before acquisition, single-cell suspensions were stained with 7-AAD sodium azide free (Miltenyi Biotec, Bergisch Gladbach, Germany) and incubated for 10 min at 4 °C in order to exclude dead cells.

### 2.3. FACS Analysis and High-Speed Cell Sorting by Flow Cytometry

Stained single-cell suspensions, obtained as described above, were immediately acquired and sorted on a FACSAriaII flow cytometer (BD Biosciences, Franklin Lakes, NJ, USA) equipped with a 488 nm blue, air-cooled argon ion laser and two photomultiplier tubes with a band pass filter of 530/30 nm (FL1), 695/40 nm (FL2). Data processing has been performed by FACS Diva Software version 6.0 (BD Biosciences).

Optimal instrument settings to gate were established by using Cytometer Setup and Tracking Beads Kit (Becton Dickinson Immunocytometry Systems, San Jose, CA, USA). Optimal drop delay was established by using Auto Drop Delay with BD AccudropTM beads (BD Biosciences).

eGFP was excited by blue laser and detected by a fluorescence 1 (FL1) filter, and 7-aminoactinomycin D (7-AAD) was excited by blue laser and detected by a fluorescence 2 (FL2) filter. Voltages for Forward-Light Scatter (FSC) and Side-Light Scatter (SSC) detectors were 385 and 330, respectively, and signals from FSC and SSC detectors were displayed by linear amplification. All observations of FL1 and FL2 were made in the log mode. A threshold of 45,000 on FSC was applied. In order to reduce abort events, particles were acquired at a rate of 1400 cells per second. The high-speed cell sorting was to be run at 70 psi pressure with a 70 μm nozzle size. The ‘‘Purity Precision Mode’’ was applied. Dead cells, which were identified by staining with 7-AAD, were excluded from the high-speed cell sorting. According to the eGFP fluorescence of the analyzed cells, two different populations were sorted. Each sorted population was harvested in a 500 uL sterile Eppendorf tube filled with 200 ul of sterile PBS.

**Figure 2:**
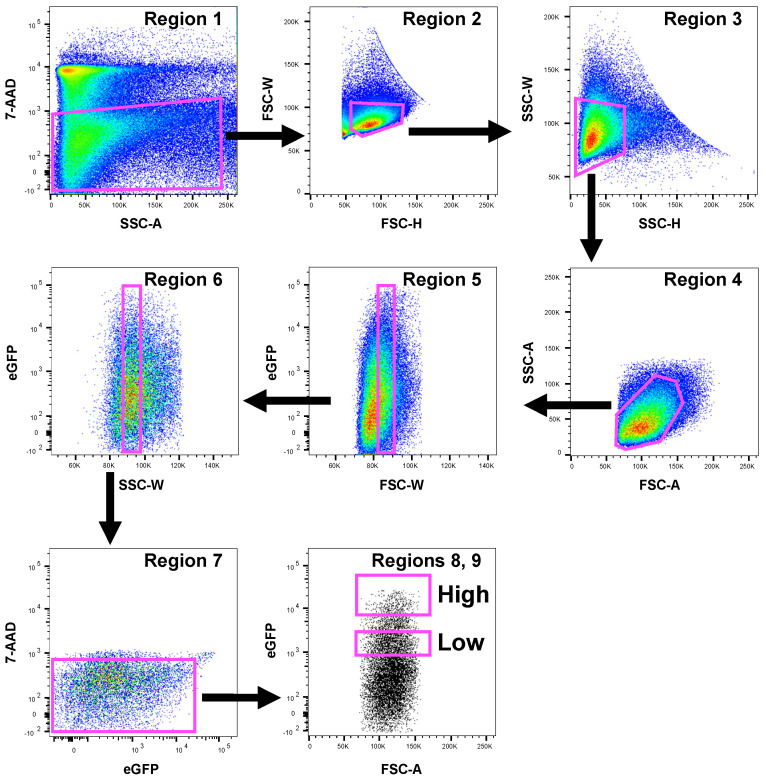
Gating strategy used to recover dopaminergic cells. Neuronal cells isolated from one representative mouse were sorted by flow cytometry and a representative gating strategy for FACS sorting to set the boundaries of eGFP+ cells is shown. First, dead cells were removed by 7-AAD vs. SSC-A plots (Region 1). Doublets were then eliminated using FSC-H versus FSC-W and SSC-H versus SSC-W plots (Regions 2 and 3). Then, cells were gated for FSC-A versus SSC-A (Region 4). Further doublet removals were performed increasing the scale for FSC-W and SSC-W versus eGFP fluorescence (Regions 5 and 6). Although eGFP shows negligible spectral overlap with 7-AAD fluorochrome, spectral overlap for 7-AAD versus eGFP was removed with Region 7. Finally, two eGFP+ cell populations were defined for sorting as High and Low (Regions 8 and 9). The axis scales for fluorescence are reported as log; the axis scales for SSC, FSC are reported as linear.

Data were analyzed using FlowJo software, Version 10.7.2 (Tree Star, Ashland, OR, USA). Detailed sorting strategy and hierarchy of the gating strategy are shown in Figure 2.

### 2.4. Cell Lysates, Reverse Transcription and Quantitative Real-Time PCR (RT-qPCR)

After sorting, cells were washed once in PBS and supernatant was completely aspirated by using a syringe and discarded, being careful to not lose the recovered cells. Power SYBR^®^ Green Cells-to-Ct™ Kit (Ambion, ThermoFisher Scientific, Monza MB, Italy) was employed, because it is suitable to quantify gene expression in a range of 10–10^5^ cells, following the manufacturer’s protocol.

Each sample was lysed using 50 μL of the Lysis Solution (49.5 µL of Kit Lysis buffer with 0.5 µL of DNase I 1 U/µL, diluted 1:100 before use), adding 1 µL of RNAse inhibitor (20 U/µL) to preserve the integrity of the total RNA. The sample was then gently mixed by pipetting up and down 5 times. The lysis reaction was carried on for 5 min at 22 °C and stopped adding 5 µL of Stop Solution. Do not wait more than 20 min before the following step. Please note that the lysates can be stored on ice up to 2 h, or frozen.

An amount of 22.5 µL of cellular lysate was reverse transcribed in a total volume of 50 µL, using 1 µM random primers of 2X SYBR^®^ RT Buffer and 20X RT Enzyme Mix. The reactions were incubated at 37 °C for 1 h, then the enzyme was inactivated at 95 °C for 5 min. Finally, 4 µL of reverted sample was employed in qPCR reactions using Power SYBR Green PCR Master Mix (containing ROX™ passive reference dye), with 200 nM of forward and reverse primers. CFX Connect Real-Time PCR Detection System (Bio-Rad Laboratories, Segrate (MI), Italy) was used for amplification.

Primers were designed with Primer3web version 4.1.0 (https://primer3.ut.ee/ 30 September 2022) [65] using mouse gene sequences deposited in the public databases (The National Center for Biotechnology Information, https://www.ncbi.nlm.nih.gov/ 30 September 2022). PCR conditions and primer sequences are reported in Table S1.

Then, the relative gene expression level was obtained as the fold change calculated using the 2^−ΔCT^ formula, in which the ΔCT represents the difference between the threshold cycle of the target gene and the reference gene, beta Actin (ACTB), for each sample analyzed.

### 2.5. Confocal Microscopy

The olfactory bulb of TH-eGFP mice was sectioned at 150 µm using a vibroslicer (WPI VSL/Campden 752), fixed in 4% paraformaldehyde/phosphate-buffered saline for at least 24 h and then collected onto gelatinized slides. Images were collected at a LSM 900 confocal microscope (Zeiss, Oberkochen, Germany) using a Plan-Apochromat 20× objective. Z-stacks of 25 μm were acquired.

### 2.6. Statistical Analysis

The Shapiro–Wilk test was used to evaluate the Gaussian distribution of the overall data. Statistical comparisons between the different groups of subjects were calculated with non-parametric analyses (Mann–Whitney non-parametric U-test or Kolmogorov–Smirnov test, when appropriated) when no Gaussian distribution was found and exact *p* values were obtained, otherwise according to their standard deviation unpaired t-students’ test was used. A *p*-value < 0.05 was considered statistically significant. Statistical analyses were performed using GraphPad Prism for Windows (version 8, GraphPad Software, San Diego, CA, USA).

## 3. Results

### 3.1. Analysis of Gene Expression of Different Population of Sorted TH-eGFP Cells from the OB

We studied the expression of factors involved in adult neurogenesis differentiation and maturation of dopaminergic cells in the mice olfactory bulb. We quantified by RT-qPCR the levels of genes differentially expressed in two groups of FACS sorted cells, hypothesized to be immature and mature dopaminergic neurons. Cells were sorted and collected on the basis of the levels of eGFP fluorescent signals in High (H-eGFP) or Low (L-eGFP) expressing neurons, respectively.

Since dopaminergic cells are characterized by specific markers, we verified their expression in the two groups, before investigating genes associated with specific pathways, related to the neurogenesis or differentiation of dopaminergic neurons. Furthermore, we analyzed other genes coding transcription factors, reported in the literature to be associated with processes of maturation of dopaminergic cells. All results are listed in Table 1, including average ± SEM of the levels of expression and *p* value.

### 3.2. Expression of the Markers of Catecholaminergic Neurons

**Figure 3:**
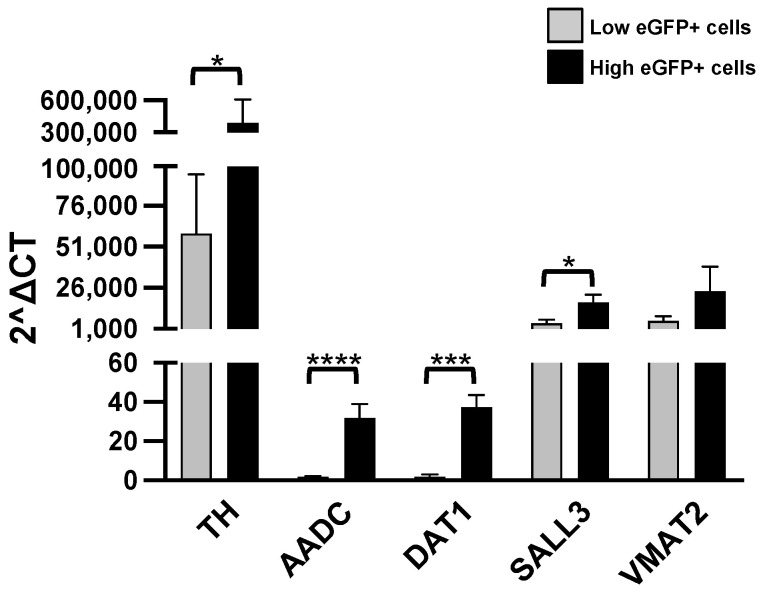
RT-qPCR analysis. Levels of the listed transcripts in the two different cell groups, L-eGFP and H-eGFP. The fold change levels were calculated using the 2^−ΔCT^ formula, and beta Actin (ACTB) as reference gene. Statistical analysis of the differences was performed as described in Materials and Methods. *p*-values < 0.05 were considered significant: * *p* < 0.05; *** *p* < 0.001; **** *p* < 0.0001.

We have first checked the reliability of our hypothesis, testing the expression of six genes, TH, AADC (aromatic L-amino acid decarboxylase), SLC6A3 (Solute Carrier Family 6 Member 3, a dopamine transporter gene, also known as DAT1), COMT (catechol-O-methyl-transferase), SALL3 (Spalt-like transcription factor 3) and VMAT2 (Vesicular Monoamine Transporter 2). Our results are illustrated in Figure 3.

As we supposed, H-eGFP cells have significantly higher levels than the L-eGFP neurons of the *TH*, *AADC*, *DAT1* and *SALL3* transcripts. On the contrary, we do not find significant differences in the expression of COMT and VMAT2 in the two groups (Table 1).

### 3.3. Expression of Genes Related to Reln Cascade

**Figure 4:**
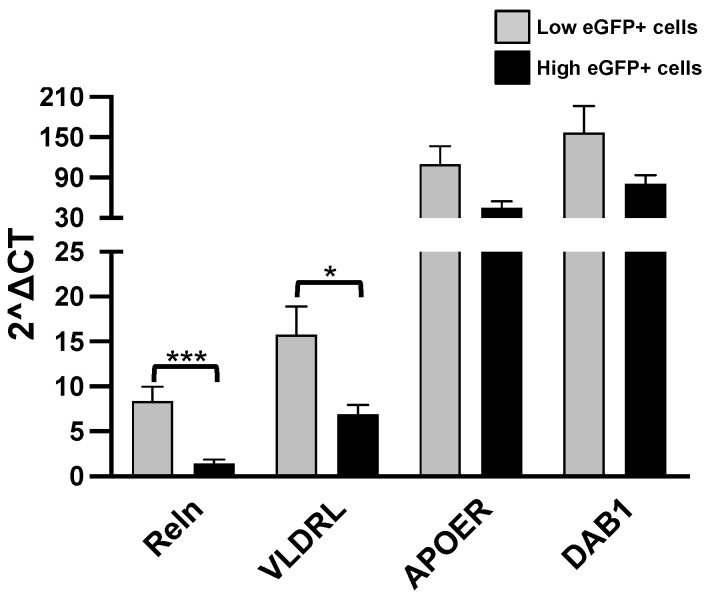
RT-qPCR analysis. Levels of the listed transcripts in the two different cell groups, L-eGFP and H-eGFP. The fold change levels were calculated using the 2^−ΔCT^ formula, and beta Actin (ACTB) as reference gene. Statistical analysis of the differences was performed as described in Materials and Methods. *p*-values < 0.05 were considered significant: * *p* < 0.05; *** *p* < 0.001.

We analyzed the expression of Reln (Reelin), ApoER2 (apolipoprotein E receptor 2), VLDLR (very-low-density lipoprotein receptor) and DAB1 (Disable homolog-1) which are all components of the Reln pathway. The results are indicated in Figure 4.

A higher expression of Reln and VLDLR was detected in the L-eGFP of neurons with respect to H-eGFP neurons, representing specific features of more immature cells. Instead, ApoER2 and DAB1 do not show significant differences.

### 3.4. Detection of Different Regulation of Transcription Factor, Controlling the Development and the Survival of Dopaminergic Neurons

**Figure 5:**
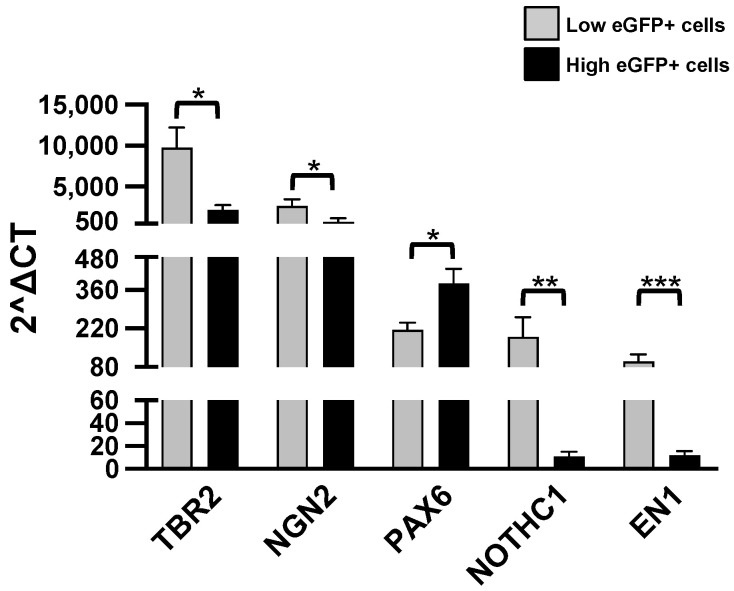
RT-qPCR analysis. Levels of the listed transcripts in the two different cell groups, L-eGFP and H-eGFP. The fold change levels were calculated using the 2^−ΔCT^ formula, and beta Actin (ACTB) as reference gene. Statistical analysis of the differences was performed as described in Materials and Methods. *p*-values < 0.05 were considered significant: * *p* < 0.05; ** *p* < 0.01; *** *p* < 0.001.

Consistently with the supposed immaturity of L-eGFP cells, we observed a higher expression of NOTCH1 (Notch Receptor 1) in L-eGFP cells (Figure 5).

On the contrary, the pleiotropic transcription factor Pax-6 (paired box 6) was increased in H-eGFP (Figure 5), consistently with their higher maturity state. We have analyzed additional transcription factors, the eomesodermin TBR2 (The T-box transcription factor Eomes), NGN2 (neurogenin 2) and EN1 (engrailed 1). We observed a higher expression of each transcript in the L-eGFP group (Figure 5).

We have checked the levels of other relevant transcription factors, DNA-binding proteins and enzymes involved in the process of neurogenesis and morphogenesis (for a complete description please refer to this comprehensive review on the topic of Pignatelli and Belluzzi [30]: ARX (Aristaless-Related Homeobox), Homeobox protein DLX1, DLX2 and DLX5 (distal-less homeobox 1, 2, 5), EGR-1 (Early Growth Response 1), FEZF1 (FEZ Family Zinc Finger 1), HES1 (hairy and enhancer of split-1), LMX1 (LIM Homeobox Transcription Factor 1 Alpha), MASH1 (mammalian achaete-scute homolog 1), MEIS2 (Meis homeobox 2), MYST4 (lysine acetyltransferase 6B), NeuroD1 (neuronal differentiation 1), TNR (tenascin-R), TSHZ1 (teashirt zinc finger family member 1), PTX3 (pentraxin-related gene), SHH (sonic hedgehog), SLIT2 (slit guidance ligand 2), GSH2 (glutathione synthetase 2), DCX (doublecortin), ZIC2 (Zic family member 2), TBR1 (T-box brain transcription factor 1), and CXCL12 (C-X-C motif chemokine ligand 12). The data, reported in Table 1, clearly show that all these genes are not significantly different in the two considered groups H-eGFP and L-eGFP. In addition, we also analyzed the levels of NURR1 (Nuclear Receptor Subfamily 4 Group A Member 2) which was found to be more expressed in the H-eGFP.

## 4. Discussion

The aim of this study is to further characterize two populations of DA interneurons found in the olfactory bulb of TH-eGFP mice. These two groups of neurons exhibit functional properties (appearance of pacemaker currents, synaptic connection with the olfactory nerve, intracellular chloride concentration, and other) marking a gradient of maturity toward the dopaminergic phenotype corresponding to the intensity of fluorescence (Pignatelli et al., 2009). To confirm these data, we set up a method to isolate them by FACS sorting and evaluated the differential expression of genes which are known to be related to immature cells or to mature dopaminergic neurons.

In L-eGFP neurons, we found genes related to the Reelin pathway while in cells expressing the higher fluorescent signal (H-eGFP neurons) these genes are found in a lower amount. The Reelin gene encodes for a glycoprotein of the extracellular matrix which plays a key role during neuronal migration and layer formation [66]. In addition, in post-mitotic neurons, Reelin regulates dendritic arborization, synaptogenesis, neurotransmitter release and excitatory modulation [67]. During development and maturation of the Central Nervous System (CNS), Reelin is involved in cortical and hippocampal layering [68,69], neuroblasts detachment [70], differentiation of radial cells [71], neurogenesis and gliogenesis processes [72]. All these processes involve the binding of Reln to its receptors: ApoEr2 and VLDLR [73,74] and the activation of its intracellular adaptor protein Disable homolog-1 (DAB1) [75,76,77,78]. In the olfactory bulb, the expression of Reelin and of its receptor ApoER2 and DAB1 have been reported in mitral cells [79]. Concerning the role of Reelin in neuroblast detachment from the RSM, its involvement has not been completely clarified since [80] identified ApoEr2 as the main receptor involved in triggering the Reelin-mediated detachment of neuroblasts while Blake et al. identified thrombospondin 1, and not Reelin, as the main ligand for ApoER2 [81]. This debate does not detract from the fact that in our study the neurons that express significantly high levels of Reelin, ApoER2, VLDLR and, although not statistically significant, DAB1 are those that have been identified in previous studies as immature cells due to their functional characteristics and low levels of eGFP immunofluorescence [46].

Consistently with this finding, we showed that L-eGFP neurons express significantly higher levels of NOTCH1, NGN2 and Trb2 than H-eGFP neurons. Reln induces detachment of neuroblasts from the chains when they arrive at the olfactory bulb [70] possibly acting in association with the NOTCH signaling. NOTCH1 cascade is activated by Reln [82,83], and it is interesting to note that both have the same trend in L-eGFP neurons. NOTCH is a single-pass transmembrane receptor controlling cell differentiation by intercellular communications among neighboring cells, and it is associated with the maintenance of proliferative state [84]. NOTCH1 is associated with cells in the SVZ, RMS, and in the postnatal brain [85,86].

NGN2 has been reported to be expressed in cells which have matured from the neuronal progenitor stage to neuronal precursors [87]. In OB, NGN2 is crucial during postnatal olfactory bulb neurogenesis [88] especially in the lineage determination of glutamatergic neurons from embryonic progenitors and adult SVZ cells [89,90,91]. Subsequently, NGN2 expression decreases, while the levels of the transcription factor TBR2 increase, indicating the switch from the neuronal precursor state to that of maturing neuroblast [88]. TRB2 is expressed in several mouse developing brain regions and associated with postmitotic and differentiating cells [92].

Finally, we quantified the levels of the homeobox protein EN engrailed 1, involved during development in survival of midbrain dopaminergic neurons [93] and specific marker of their phenotype and associated with DA neurons in the OB [94]. We found that also the expression of EN1 is higher in L-eGFP neurons. Based on these results, the expression of NOTCH1, NGN2, Trb2, and EN1 in L-eGFP neurons indicate that these are immature cells set to become dopaminergic neurons.

In this study, we also analyzed the expression of the paired box gene 6 (PAX6). Pax-6 controls cell adhesion molecules, short-range cell-to-cell signaling molecules, playing a role in key biological processes including cell proliferation and migration. It has been found in the embryonic LGE (lateral ganglionic eminence) and the postnatal SVZ, the RMS, while in the adult brain, Pax6 is expressed in the OB in the granule cell layer (GCL) and the glomerular layer [95,96], suggesting that it may be involved in adult neurogenesis. Neuronal precursors PAX6 is necessary for their specification to dopaminergic neurons. Indeed, PAX6 mutant mice produce progenitors that evolve in neuroblasts that can migrate into the OB but fail to generate dopaminergic periglomerular interneurons [97]. Thus, having found that PAX6 mRNA is highly expressed in H-eGFP neurons, but not in L-eGFP cells, we can hypothesize that the former neurons are mature DA periglomerular cells. To support this hypothesis, we analyzed the expression of genes that are specific for mature dopaminergic neurons.

TH and AADC are an ineludible choice, as they are markers typically characterizing all catecholaminergic neurons, which in OB are only dopaminergic cells. These data are supported by the literature in which it has been demonstrated that their mRNA and protein cannot be detected during development [48,98]. The expression of DAT1 and VMAT2 is correlated to a dopaminergic phenotype. We did not observe any differential expression for the vesicular monoamine transporter VMAT2, making us hypothesize that H-eGFP neurons might correspond to the non-exocytotic dopaminergic population of the OB which may accumulate dopamine and release it constitutively [99]. Among the genes essential for the determination of the dopaminergic phenotype, we analyzed the expression of SALL3, a zinc finger transcription factor necessary for the terminal differentiation and maturation of TH-expressing cells [100] and for the correct formation of the glomerular layer of the olfactory bulb. In Sall3-null mice a total loss of dopaminergic neurons is observed in the olfactory bulb, while, in wild type animals, Sall3 expression strongly correlates with the expression of TH [48,101]. Sall3 expression is linked with DA phenotypic differentiation during development [48]. Moreover, Sall3 deficiency determines the development of an hypocellular glomerular layer due to a decreased number of interneurons, accompanied by a complete absence of TH-positive cells [102]. These data correlate with the finding that Sall3 induces the transcription activity of the proximal promoter of the TH gene [101].

We also analyzed DCX marker that it was reported be highly expressed in neuronal precursors in the early phases of neurogenesis occurring in the SVZ, and it is necessary for immature cell migration [103,104], this gene it becomes largely down-regulated along the path toward the OB, progressively as cells develop a more mature phenotype [105]. Consistently, our data show that the expression of DCX mRNA is low in the OB, and that the difference in the two cellular subpopulations we examined is not significant. This is probably due to the fact that DCX is no more necessary for the migration of these cells. This is consistent with our observations. In the adult mouse olfactory bulb, DCX protein might be expressed in neurons with the morphology of adult neurons in the glomerular and granular layers, where it is believed to participate in plasticity mechanisms in adult neurons [106].

In addition, we also analyzed the expression levels of COMT (catechol-O-methyl-transferase) since it has been reported in the literature that contrary to what happens in most dopaminergic systems where the vesicular release of DA is cleared by the dopamine transporter (DAT), and the OB enzymatic catalysis occurs via catechol-O-methyl-transferase (COMT) which predominate over DAT re-uptake [107]. The expression levels of COMT for the two groups of cells H-eGFP versus L-eGFP are, however, not significantly different in our analysis. These data are possibly due to the fact that we are analyzing isolated cells and not the complete circuit. Indeed, COMT has been suggested to be expressed in neurons with which dopaminergic neurons make synapse [108].

## 5. Conclusions

In conclusion, the data described in this manuscript support the fact that the two populations of neurons expressing low- or high-fluorescent eGFP belong to neurons at different stages of development. The FACS method set up to isolate them can be used not only for further characterizing these cells but to engineer new neurons for therapeutic approaches such as transplantation into specific areas of the brain affected by Parkinson’s disease.

## Figures and Tables

**Table 1:** Gene expression value for GFP cell groups.

Gene	Average ± of Gene Expression of L-GFP Group, [N]	Average ± of Gene Expression of H-GFP Group, [N]	*p*-Value
**** AADC **(aromatic L-amino acid decarboxylase)**	1.87 ± 0.36, [14]	31.95 ± 6.94, [23]	**<0.0001**
ApoEr2 apolipopritein E receptor 2	109.90 ± 26.48, [19]	45.26 ± 9.27, [18]	0.13
ARX aristaless-related homeobox	289.80 ± 123.40, [9]	131.10 ± 44.53, [9]	0.6
COMT catechol-O-methyltransferase	1.70 ± 0.29, [3]	1.99 ± 0.51, [3]	0.66
CXCL12 C-X-C motif chemokine ligand 12	0.09 ± 0.08, [8]	0.007 ± 0.002, [8]	0.59
DAB1 DAB adaptor protein 1	156.80 ± 39.59, [17]	80.62 ± 12.95, [20]	0.22
*** DAT **dopamine membrane transporter**	1.93 ± 1.09, [4]	37.48 ± 6.06 [8]	**0.0005**
DCX doublecortin	26.96 ± 11.76, [11]	60.91 ± 22.82, [15]	0.33
DLX1 distal-less homeobox 1	23.25 ± 5.12, [22]	17.66 ± 4.80, [19]	0.57
DLX2 distal-less homeobox 1	23.79 ± 5.63, [13]	13.14 ± 3.01, [13]	0.10
DLX5 distal-less homeobox 5	40.30 ± 10.75, [13]	18.73 ± 3.85, [13]	0.26
EGR1 early growth response 1	63.11 ± 48.14, [13]	144.6 ± 98.11, [14]	0.62
*** EN1 **engrailed 1**	99.94 ± 25.34, [13]	12.04 ± 3.69, [12]	**0.0003**
FEZF1 FEZ family zinc finger 1	24.53 ± 13.74, [4]	10.52 ± 4.39, [7]	0.25
GSH2 glutathione synthetase 2	26.99 ± 7.22, [19]	15.67 ± 4.02, [14]	0.70
HES1 hairy and enhancer of split 1	24.34 ± 7.18, [11]	14.49 ± 3.46, [12]	0.22
LMX1A LIM homeobox transcription factor 1 alpha	32.30 ± 14.26, [12]	177.2 ± 80.04, [12]	0.11
MASH1-ASCL1 achaete-scute family bHLH transcription factor 1	5.91 ± 1.03, [21]	11.67 ± 3.37, [20]	0.27
MEIS2 Homeobox protein Meis2	134.8 ± 30.79, [11]	350.30 ± 103.90, [14]	0.34
MYST-4 K(lysine) acetyltransferase 6B	68.78 ± 18.46, [15]	30.03 ± 5.69, [15]	0.40
NEUROD1 neuronal differentiation 1	1328.00 ± 860.6, [10]	2650 ± 1205, [13]	0.48
* NGN2 **neurogenin 2**	2668 ± 824.70, [11]	717.80 ± 452.8 [10]	**0.036**
** NOTCH1 **notch receptor 1**	189.6 ± 71.15, [16]	10.79 ± 4.41, [14]	**0.0079**
* NURR1 **Nuclear Receptor Subfamily 4 Group A Member 2**	16.78 ± 2.72, [8]	34.68 ± 6.56, [9]	**0.02**
* PAX6 **paired box gene 6**	214.6 ± 26.74, [7]	384.6 ± 52.59, [7]	**0.01**
PTX3 pentraxin-related gene	14.80 ± 3.49, [11]	16.39 ± 6.47, [13]	0.46
*** Reln **Reelin**	8.37 ± 1.59, [16]	1.42 ± 0.43, [9]	**0.0006**
* SALL3 **spalt-like transcription factor 3**	4263.00 ± 2151.00, [11]	17,117 ± 4625, [16]	**0.0109**
SHH sonic hedgehog	48.95 ± 14.28, [11]	100.9 ± 28.88, [17]	0.46
SLIT2 slit guidance ligand 2	7.60 ± 2.09, [19]	6.48 ± 1.29, [10]	0.78
TBR1 T-box brain transcription factor 1	1325 ± 487.8, [11]	1356 ± 679.5, [8]	0.27
* TBR2 **T-box brain transcription factor 1**	9768 ± 2452, [15]	2200 ± 563.6, [13]	**0.04**
* TH **tyrosine hydroxylase**	59,131 ± 36,008, [21]	392,278 ± 217,515, [22]	**0.0268**
TNR tenascin-R	58.34 ± 16.92, [15]	20.32 ± 5.98, [11]	0.20
* VLDR **very-low-density lipoprotein receptor**	15.75 ± 3.16, [17]	6.91 ± 1.00, [18]	**0.032**
VMAT2 vesicular dopamine transporter	5915 ± 2789, [6]	24,002 ± 14,880, [5]	0.33

Statistically significant values are shown in bold. *p*-values < 0.05 were considered significant: * *p* < 0.05; ** *p* < 0.01; *** *p* < 0.001; **** *p* < 0.0001.

## Data Availability

Data are contained within this article and the Supplementary Material.

## Supplementary Materials

The following supporting information can be downloaded at: https://www.mdpi.com/article/10.3390/biology12030367/s1, Table S1: Primers and conditions used for amplification reactions.
