# Supplementary material for: Characterization by Gene Expression Analysis of Two Groups of Dopaminergic Cells Isolated from the Mouse Olfactory Bulb
Source: Biology (Basel). 2023 Feb 25;12(3):367. doi: 10.3390/biology12030367 (PMC10045757; doi:10.3390/biology12030367)
Supplement: Supplementary file 1 [file biology-12-00367-s001.zip › biology-2164823-supplementary.pdf]

Supplementary table S1. Primers and conditions used for amplification reactions.

Primer name	Primer sequence	First step of denaturation cycles melting/elongation
AADCFF	5'-ATTTGCACAGAAGTCATTC-3'	50°C 2' - 95°C 10'
		50 cycles:
AADCFR	5'-TTTGTAAGAGAGTTTCGTTC-3'	95°C 20''- 60°C 1'
ACTBF	5'-GTGGGCCGCTCTAGGCACCA-3'	50°C 2' - 95°C 10'
		50 cycles:
ACTBR	5'-CGGTTGGCCTTAGGGTTCAGGGGG-3'	95°C 20''- 60°C 1'
ApoEr2F	5'-CCTCAGTCTACCTCAACTAC-3'	50°C 2' - 95°C 10'
		50 cycles:
ApoEr2R	5'-CTCTGTGCTATGGTTCTGT-3'	95°C 20''- 60°C 1'
ARXF	5'-CAATCAGTACCAGGAAGAG-3'	50°C 2' - 95°C 10'
		50 cycles:
ARXR	5'-AGTAGGAGGAGAGCAAAG-3'	95°C 20''- 60°C 1'
COMTF	5'-GGGTGACGCAAAAGGCCAAA-3'	50°C 2' - 95°C 10'
		45 cycles:
COMTR	5- ATTGTCAGCTAGGAGCACCG-3'	95°C 20''- 60°C 1'
CXCL12F	5'- CAAGGTCGTCGCCGTGC-3'	50°C 2' - 95°C 10'
		45 cycles:
CXCL12R	5'-GTAGCTCAGGCTGACTGGTTT-3'	95°C 20''- 60°C 1'
DAB1F	5'-CATCTACCAACTCACCTC-3'	50°C 2' - 95°C 10'
		50 cycles:
DAB1R	5'-GTGCTTCTTGTTCTTCAC-3'	95°C 20''- 60°C 1'
DAT1F	5'-TCCTGCGTGGAGTCACCCTCC-3'	50°C 2' - 95°C 10'
		45 cycles:
DAT1R	5'-ACCCAACGCCAAGGGAGAAGC-3'	95°C 20''- 60°C 1'
DCXF	5'-CAAAAGACATCTGCTAAAAG-3'	50°C 2' - 95°C 10'
		50 cycles:
DCXR	5'-GTGTAGAGATAGGAGACTGC-3'	95°C 20''- 60°C 1'
DLX1F	5'-AACTCAGTACTTAGCTCTGC-3'	50°C 2' - 95°C 10'
		50 cycles:
DLX1R	5'-AGCTTCTTGAACTTGGAG-3'	95°C 20''- 60°C 1'

DLX2F	5'-TCCAGAAGACCCAGTATC-3'	50°C 2' - 95°C 10'
		50 cycles:
DLX2R	5'-TTCCACATCTTCTTGAAC-3'	95°C 20''- 60°C 1'
DLX5F	5'-CTTTACAGAGAAGGTTTCAG-3'	50°C 2' - 95°C 10'
		50 cycles:
DLX5R	5'-GATCTTGGATCTTTTGTTC-3'	95°C 20''- 60°C 1'
EGR1F	5'-TACCAAAATCCATTTAAGAC-3'	50°C 2' - 95°C 10'
		50 cycles:
EGR1R	5'-GATGGGTAAGAAGAGAGTG-3'	95°C 20''- 60°C 1'
EN1F	5'-TGGTCAAGACTGACTCAC-3'	50°C 2' - 95°C 10'
		50 cycles:
EN1R	5'-TCGTTCTTTTTCTTCTTTAG-3'	95°C 20''- 60°C 1'
FEZF1F	5'-CCACGTTAAACACACATAC-3'	50°C 2' - 95°C 10'
		50 cycles:
FEZF1R	5'-AACTTGTGATTTTTGTAATTC-3'	95°C 20''- 60°C 1'
GSH2F	5'-TCTTCCAATATGTACCTGTC-3'	50°C 2' - 95°C 10'
		50 cycles:
GSH2R	5'-GTTCTGAAACCAGATTTTC-3'	95°C 20''- 60°C 1'
HES1F	5'-ACTGAAAACACTGATTTTG-3'	50°C 2' - 95°C 10'
		50 cycles:
HES1R	5'-AGTCATTTCCAGAATGTC-3'	95°C 20''- 60°C 1'
LMX1AF	5'-CGTCTACAACTCTGATCC-3'	50°C 2' - 95°C 10'
		50 cycles:
LMX1AR	5'-CAGGTTACTGAGAGATGTG-3'	95°C 20''- 60°C 1'
MASH1-ASCL1F	5'-AACTACTCCAACGACTTG-3'	50°C 2' - 95°C 10'
		50 cycles:
MASH1-ASCL1R	5'-CAGAACCAGTTGGTAAAG-3'	95°C 20''- 60°C 1'
MEIS2F	5'-TGGGAGAAGTCTCCTTGGTTTGG-3'	50°C 2' - 95°C 10'
		50 cycles:
MEIS2R	5'-AATGGGGCAGCTCATCGTACC-3'	95°C 20''- 60°C 1'
MYST-4F	5'-GAAAAGATGTAGATGATGATG-3'	50°C 2' - 95°C 10'
		50 cycles:
MYST-4R	5'-ACACTTGTCTGCACTTTAG-3'	95°C 20''- 60°C 1'
NEUROD1F	5'-TCAGCATCAATGGCAACTTC-3'	50°C 2' - 95°C 10'
		50 cycles:
NEUROD1R	5'-AAGATTGATCCGTGGCTTTG-3'	95°C 20''- 60°C 1'

NGN2F	5’-TCATCCTCCAACTCCACGTC-3’	50°C 2’ - 95°C 10’
		50 cycles:
NGN2R	5’-GCTGCCAGTAGTCCACGTC-3’	95°C 20’’- 60°C 1’
NOTCH1F	5’-CAGAACTTACAGCTCCAG-3’	50°C 2’ - 95°C 10’
		50 cycles:
NOTCH1R	5’-GTTGTACATCTGCCTGAC-3’	95°C 20’’- 60°C 1’
NURR1F	5’-ACTTGCAGAATATGAACATC-3’	50°C 2’ - 95°C 10’
		50 cycles:
NURR1R	5’-ATTTTGTTTTGTAGCTCTTC-3’	95°C 20’’- 60°C 1’
PAX6F	5’-CCTGTCTCCTCCTTCACATCAG-3’	50°C 2’ - 95°C 10’
		50 cycles:
PAX6R	5’-TTGGTGAGGGCGGTGTCT-3’	95°C 20’’- 60°C 1’
PTX3F	5’-GAGGAATCGCTACCCTGACA-3’	50°C 2’ - 95°C 10’
		50 cycles:
PTX3R	5’-GGGTACACCTCCTCGTAGGG-3’	95°C 20’’- 60°C 1’
RelnF	5’-ATGAAAGGAGTTCTACTGC-3’	50°C 2’ - 95°C 10’
		50 cycles:
RelnR	5’-TCATCATGTAATTTTGTTTG-3’	95°C 20’’- 60°C 1’
SALL3F	5’-ACCAAGGGCAATCTCAAGG-3’	50°C 2’ - 95°C 10’
		50 cycles:
SALL3R	5’-ATGGGGTTCTCCACAGACAG-3’	95°C 20’’- 60°C 1’
SHHF	5’-ATTCAGAGGAGTCTCTACAC-3’	50°C 2’ - 95°C 10’
		50 cycles:
SHHR	5’-CTTTGGATTCATAGTAGACC-3’	95°C 20’’- 60°C 1’
SLIT2F	5’-GCCTGTCAAACAACTAAG-3’	50°C 2’ - 95°C 10’
		50 cycles:
SLIT2R	5’-CACTTTCTCAACCTCGTC-3’	95°C 20’’- 60°C 1’
TBR1F	5’-CAAACAACAATGGGCAGATG-3’	50°C 2’ - 95°C 10’
		50 cycles:
TBR1R	5’-TCTCCGGAAAAGTGAACGTC-3’	95°C 20’’- 60°C 1’
TBR2F	5’-GCACATCGTGGAAGTGACAG-3’	50°C 2’ - 95°C 10’
		50 cycles:
TBR2R	5’-GGGTGATATCCGTGTTTTGG-3’	95°C 20’’- 60°C 1’
ThFF	5'-GCAGTGCCAGAGAGGACAAG-3'	50°C 2’ - 95°C 10’
		50 cycles:
ThFR	5-GGCAGGCATGGGTAGCATAG-3'	95°C 20’’- 60°C 1’

TNRF	5'-CAGTCTTTGCCTACTATGAC-3'	50°C 2' - 95°C 10'<br>50 cycles:
TNRR	5'-CAACATCATTGTCTCTGTC-3'	95°C 20''- 60°C 1'
VLDRF	5'-GTATCAGAAGTCAGTGTTCC-3'	50°C 2' - 95°C 10'<br>50 cycles:
VLDRR	5'-ATTCCTCCACATCAAGTAG-3'	95°C 20''- 60°C 1'
VMAT2F	5'-GCTATGGCCCTGAGCGATCTG-3'	50°C 2' - 95°C 10'<br>50 cycles:
VMAT2R	5'-AGCTGGGAATGATGGGAACTACG-3'	95°C 20''- 60°C 1'
